# DnaJB6: an indirect interaction protein of 15-kDa selenoprotein

**DOI:** 10.1186/1750-1326-7-S1-O11

**Published:** 2012-02-07

**Authors:** Jing Tian, Qiong Liu, Jiazuan Ni

**Affiliations:** 1 College of Life Sciences, Shenzhen University, Shenzhen 518060, P.R.China

## Background

15-kDa selenoprotein (Sep15) is a protein containing seleno-cysteine (Sec) encoded by UGA, a stop codon. Sep15 is possibly involved in glycoprotein folding, ER stress and redox homeostasis. It is reported that Sep15 mRNA was detected in brain and a ^75^Se labeled protein dot was also appeared at the corresponding position of Sep15 (molecular weight= ~15-kDa, pI value: 4.6-4.8). But the function of Sep15 in brain is unknown. DnaJB6 is a molecular chaperone highly enriched in the central nervous system. It inhibits Huntingtin aggregation and toxicity independently and has been found to be present in core of lewy bodies in Parkinson’s disease and highly up-regulated in Parkinsonian astrocytes.

## Method

cDNA coding mature Sep15 was cloned and the codon ‘TGA’ coding for Sec was mutated into ‘TGC’ coding for cystein (Cys) (Because no selenoprotein synthesis system was discovered in yeast, ‘TGA’ should be mutated into ‘TGC’ to use yeast two hybridization to screen selenoprotein interaction protein). The cDNA was cloned into bait vector of yeast two hybridization system. The interaction protein of Sep15 was screened in human fetal brain library according to the manual (Clontech, U.S.A). The interaction between Sep 15 and the prey protein was confirmed by FRET and co- immunoprecipitation (co-IP).

## Result

As a result, DnaJ/Hsp40 homologue, subfamily B, member 6 (DnaJB6) was screened out as well as an unknown protein. The cDNA sequence coding for the unknown protein could be amplified by PCR but not be sequenced. The data of FRET showed that Sep15 interacted with YFP but not interacted with DnaJB6. It implied that there is possibly another protein contained between Sep15 and DnaJB6. The results of coIP showed that Sep15 and DnaJB6 could be coprecipitated by antibody. It is probably that Sep15, DnaJB6 and an unknown protein form a complex to play some roles in Huntingtin’s disease (HD) and Parkinson’s disease (PD).

## Conclusion

DnaJB6 and an unknown protein were screened out as the interaction proteins of Sep15; results of RRET and coIP showed that DnaJB6 probably interacted with Sep15 indirectly.

